# Prognostic and predictive role of [^18^F]fluorodeoxyglucose positron emission tomography (FDG‐PET) in patients with unresectable malignant pleural mesothelioma (MPM) treated with up‐front pemetrexed‐based chemotherapy

**DOI:** 10.1002/cam4.1182

**Published:** 2017-09-21

**Authors:** Paolo Andrea Zucali, Egesta Lopci, Giovanni Luca Ceresoli, Laura Giordano, Matteo Perrino, Gianluigi Ciocia, Letizia Gianoncelli, Elena Lorenzi, Matteo Simonelli, Fabio De Vincenzo, Lucia Rebecca Setti, Cristiana Bonifacio, Maria Bonomi, Emilio Bombardieri, Arturo Chiti, Armando Santoro

**Affiliations:** ^1^ Oncology Humanitas Clinical and Research Hospital Rozzano (Milan) Italy; ^2^ Nuclear Medicine Humanitas Clinical and Research Hospital Rozzano (Milan) Italy; ^3^ Oncology Humanitas Gavazzeni Clinic Bergamo Italy; ^4^ Biostatistics Humanitas Clinical and Research Hospital Rozzano (Milan) Italy; ^5^ Nuclear Medicine Humanitas Gavazzeni Clinic Bergamo Italy; ^6^ Radiology Humanitas Clinical and Research Hospital Rozzano (Milan) Italy; ^7^ Humanitas University Rozzano Milan Italy

**Keywords:** Chemotherapy, FDG‐PET, malignant pleural mesothelioma, predictive role, prognostic role

## Abstract

The aim of this study was to evaluate the role of metabolic parameters analyzed at baseline and at interim FDG‐PET in predicting disease outcome in unresectable MPM patients receiving pemetrexed‐based chemotherapy. A consecutive series of MPM patients treated between February 2004 and July 2013 with first‐line pemetrexed‐based chemotherapy, and evaluated by FDG‐PET and CT scan at baseline and after two cycles of chemotherapy, was reviewed. Best CT scan response was assessed according to modified RECIST criteria. Progression‐free survival (PFS) and overall survival (OS) were correlated with FDG‐PET parameters, such as maximum standardized uptake value (SUV_max_), total lesion glycolysis (TLG), and percentage changes in SUV_max_ (∆SUV) and TLG (∆TLG). Overall, 142 patients were enrolled; 77 (54%) received talc pleurodesis before chemotherapy. Baseline SUV_max_ and TLG showed a statistically significant correlation with PFS and OS (*P* < 0.05) in both group of patients (treated and untreated with pleurodesis). In 65 patients not receiving pleurodesis, SUV_max_ reduction ≥25% (∆SUV ≥ 25%) and TLG reduction ≥30% (∆TLG ≥ 30%) were significantly associated with longer PFS (*P* < 0.05). Patients showing both ∆SUV ≥ 25% and ∆TLG ≥ 30% responses had a significant reduction in the risk of disease progression (HR:0.31, *P* < 0.001) and death (HR:0.52, *P* = 0.044). Neither ∆SUV nor ∆TLG showed similar association with survival outcomes in patients treated with pleurodesis. Our study confirmed the prognostic role of baseline FDG‐PET in a large series of MPM patients treated with first‐line pemetrexed‐based chemotherapy. Moreover, use of ∆SUV ≥ 25% and ∆TLG ≥ 30% as cut‐off values to define early metabolic response supported the role of FDG‐PET in predicting disease outcome and treatment response in patients not receiving pleurodesis.

## Introduction

Malignant pleural mesothelioma (MPM) is a rare and mostly fatal tumor, whose incidence is unfortunately increasing worldwide 1. At diagnosis, the majority of MPM patients are not amenable to up‐front radical surgery; thus chemotherapy represents the standard treatment option. Proper definition of baseline prognostic characteristics and reliable assessment of response to therapy are important components of patient care in everyday practice as well as in clinical trials. However, tumor assessment and response evaluation with conventional criteria based on contrast‐enhanced computed tomography (CT) measurements are challenging in MPM, because of its diffuse pattern of growth. Modified RECIST criteria have been implemented and are considered the reference standard in clinical practice and ongoing trials. However, they have a high interobserver variability and were not supported by theoretical studies on modeling of mesothelioma growth 2, 3, 4, 5. Moreover, like all CT criteria, they do not take into account the viability of tumor tissue, which can be better assessed with a functional imaging technique such as [18F]fluorodeoxyglucose positron emission tomography (FDG‐PET) 3, 6.

Prognostic scores based on clinical factors, such as histological subtype, gender, Eastern Cooperative Oncology Group (ECOG) performance status (PS), and leukocyte and platelet counts have been proposed and validated by the Cancer and Leukemia Group B (CALGB) and the European Organization for Research and Treatment of Cancer (EORTC) 7, 8. The tumor avidity for FDG has been investigated as a surrogate marker of tumor biology. Nowak et al. incorporated semiquantitative PET parameters and pleurodesis into pretreatment predictors, proposing a prognostic nomogram 9. More recently, other authors have confirmed that pretreatment FDG‐PET data are robust predictors of survival in MPM, with volume‐based PET parameters and histology being the main independent prognostic factors 10, 11, 12.

Other studies have explored the value of FDG uptake in response evaluation during chemotherapy. In fact, the early identification of responders to chemotherapy should make possible to avoid ineffective treatment with significant toxicities in these patients, usually elderly, with several comorbidities and reduced performance status, allowing also the optimization of the economic resources of the public health system. Different PET parameters were taken into account when analyzing the metabolic response (MR), defined as a decrease in the maximum standardized uptake value (SUV_max_), or with dedicated algorithms analyzing volume‐based parameters, such as total glycolytic volume (TGV) or total lesion glycolysis (TLG) 11, 12, 13, 14, 15, 16, 17, 18. All these studies, although conducted in small patient cohorts, suggested that in MPM patients treated with chemotherapy, an early reduction in FDG uptake could be significantly correlated with outcome, especially when talc pleurodesis is not performed at diagnosis.

The aim of this study was to evaluate the role of FDG‐PET parameters in predicting disease outcome in a larger cohort of patients with MPM patients treated with up‐front pemetrexed‐based chemotherapy.

## Materials and Methods

### Study population

A consecutive series of MPM patients treated in our Institutions (Humanitas Clinical and Research Center, Rozzano, Milan, Italy and Humanitas Gavazzeni Clinic, Bergamo, Italy) between February 2004 and July 2013 with up‐front pemetrexed‐based chemotherapy, and evaluated by FDG‐PET and CT scan at baseline and after two cycles of therapy, were retrospectively assessed.

Patients who received pleurodesis were included in our study, whereas patients who received less than two cycles of chemotherapy were excluded. Eligibility criteria comprised age ≥18 years, a histological diagnosis of MPM, ECOG PS ≤2, and an estimated life expectancy >12 weeks. The EORTC prognostic score for MPM (good vs. poor) was calculated for each patient 8.

Treatment was repeated for a maximum of six cycles, or until progression or unacceptable toxicity. After completion of chemotherapy, patients were evaluated with chest–abdomen CT scans every 3 months until disease progression. Patients were also followed up for survival until death, or last contact if still alive. This study was conducted with the approval of the local ethics committee, and according to the Helsinki Declaration. The trial was registered at www.clinicaltrials.gov (NCT00969098).

### Imaging modalities

Imaging modalities have been described previously 19. Chest–abdomen CT scans were acquired with a Philips Aura single‐slice system in the first 22 patients, and with either Philips Brilliance or Philips Mx 8000 16 scanners in the following cases. PET scans were obtained from the base of the skull to the thighs using a Siemens ECAT ACCEL full‐ring scanner until February 2007 (*n* = 27), whereas later images were acquired on an integrated PET/CT tomograph: (A) Siemens Biograph LSO 6 scanner, with an integrated 6‐slice CT; (B) GE Discovery PET/CT 690, with an integrated 64‐slice CT; (C) Phillips Gemini LXL PET/CT with an integrated 16‐slice CT. In order to ensure consistent semiquantitative and quantitative values, each patient was studied during the course of the therapeutic protocol with the same PET or PET/CT scanner. Moreover, since 2011 all our tomographs were accredited with the EANM Research Ltd (EARL) program and image analysis was performed using standardized algorithms 20.

Tumor burden was calculated with three‐dimensional volumes of interest (VOIs) drawn on the volume of metabolic tumor‐related activity. The standard method of quantification was performed as described by Boucek et al. in the first 29 patients (in whom volume‐based analysis was done by a semiautomated iterative threshold‐based region‐growing algorithm developed at Sir Charles Gairdner Hospital in Nedlands, Australia), whereas in the remaining patients the analysis for TLG computation was done using liver‐based threshold semiautomated contouring on the GE ADW4.6 workstation (GE Healthcare, Waukesha, WI) 14, 21. Two board‐certified nuclear medicine physicians used independently, and blinded to each other, the three‐dimensional volume‐based region‐growing algorithm or the new liver‐based quantitative analysis method in the same patients 19. We previously evaluated the consistency between the two techniques: the three‐dimensional volume‐based region‐growing algorithm and the new liver‐based quantitative analysis method 22. Both methods defined VOIs at baseline and interim scans, corresponding to the metabolic tumor volume (MTV), while the semiquantitative measures of SUV_max_ and SUV_mean_ were obtained from the tissue within the VOI: SUV_max_ was defined as the highest pixel value and SUV_mean_ was defined as mean SUV related to the tumor burden. Calculation of TLG was done according to the following formula: MTV (ml) × SUV_mean_ = TLG.

### Response assessment

Response assessment methods have been previously described 19. Modified RECIST criteria were used to classify tumor response to treatment as complete response (CR), partial response (PR), stable disease (SD), or progressive disease (PD) 2.

Tumor metabolic response with FDG‐PET was based on measurements obtained at the same time‐point as for interim CT scan (at baseline and after two cycles of chemotherapy) according to two different parameters: (A) percentage change in SUV_max_ between baseline and interim PET (∆SUV); (B) percentage change in TLG between baseline and interim PET (∆TLG). In both cases, data were analyzed in continuous form, applying cut‐off percentages of metabolic response obtained by merging previously published data from our hospital. Dedicated statistical analyses of this study cohort were also performed 23, 24.

### Statistical analyses

This was an observational retrospective analysis on a consecutive series of MPM patients, stratified according to previous talc pleurodesis. Patient characteristics were described in terms of number and percentage, or median and range. For continuous data, differences between groups were compared by Student's *t* test or the Wilcoxon test, when appropriate.

Progression‐free survival (PFS) was defined as the time from the first day of chemotherapy treatment until progression, death from any cause or the last visit when a patient was alive without progression. Overall survival (OS) was defined as the time between the start of treatment and patient death or last contact for patients who were alive.

Survival curves were generated with the Kaplan–Meier method. Statistically significant variables in the univariate analysis were included in the multivariate model if they confirmed an independent effect. Hazard ratios (HR) with 95% confidence intervals (CI) were calculated with the Cox proportional‐hazards regression model in univariate and multivariate analyses. For continuous variables, in the case of a statistically significant association, a recursive regression tree was estimated in order to identify a cut‐off value to discriminate patients into different prognostic groups. Statistical significance was set at *P* < 0.05 for each evaluation.

All analyses were performed using R software, version 3.0.3 (R Foundation for Statistical Computing, Vienna, Austria); graphics were made using Stata Statistical Software, version 13 (StataCorp. 2013., College Station, TX).

## Results

One hundred and forty‐two patients fulfilling the study inclusion criteria were considered for the analysis. With a median follow‐up of 45.2 months (IQR: 26.9; 64.8), the median PFS (mPFS) and median OS (mOS) were 7.6 (IQR: 4.5; 13.7) and 14.5 (IQR: 8.1; 28.5) months, respectively. According to modified RECIST criteria, PR was observed in 51 (33.8%), SD in 78 (51.7%), and PD in 22 (14.6%) patients. Patient characteristics are listed in Table 1. PET parameters distribution stratified for talc pleurodesis is reported in Table 2.

**Table 1:** Baseline patient characteristics

Characteristics	All		No talc pleurodesis		Talc pleurodesis	
	No./median	%/range	No./median	%/range	No./median	%/range
All	142	100	65	45.8	77	54.2
Gender						
Male	94	66.2	37	56.9	57	74.0
Female	48	33.8	28	43.1	20	26.0
ECOG PS						
0	86	60.6	35	53.9	51	66.2
1–2	55	38.7	29	44.6	26	33.8
Unknown	1	0.7	1	1.5		
Histology						
Epithelioid	116	81.7	52	80.0	64	83.1
Other	25	17.6	12	18.5	13	16.9
Unknown	1	0.7	1	1.5		
Type of chemotherapy						
CBDCA‐PEM	112	78.9	49	75.4	63	81.8
CBDCA‐PEM‐BEVA	27	19.0	15	23.1	12	15.6
CDDP‐PEM	1	0.7	1	1.5	0	0
PEM	2	1.4	0	0	2	2.6
N of cycles of chemotherapy	6	2;9	6	2;9	6	2;9
EORTC score						
Good	81	57.0	32	49.2	49	63.6
Poor	60	42.3	32	49.2	28	36.4
Unknown	1	0.7	1	1.5		

ECOG, Eastern Cooperative Oncology Group; PS, performance status; CBDCA‐PEM, Carboplatin and pemetrexed; CBDCA‐PEM‐BEVA, Carboplatin, pemetrexed, and bevacizumab; CDDP‐PEM, Cisplatin and pemetrexed; EORTC, European Organisation for Research and Treatment of Cancer.

**Table 2:** PET parameters distribution stratified for talc pleurodesis

Marker	No Talc pleurodesis		Talc pleurodesis	
	Median	Range	Median	Range
SUV_max_ at baseline	6.8	1.8;23.9	7.6	0;25.3
∆SUV (%)	−13.8	−100;166	0	−55.1;167.3
TLG at baseline	601.7	0.64;10472.5	423.4	0;14288.3
∆TLG (%)	−47	−100;841.2	−37.9	−97.4;946.9

SUV_max_, maximum standardized uptake value; ∆SUV, percentage change in SUV_max_ between baseline PET and interim PET after two cycles of therapy; TLG, total lesion glycolysis; ∆TLG; percentage change in TLG between baseline PET and interim PET after two cycles of therapy.

### Patients not treated with talc pleurodesis

*S*ixty‐five patients did not receive talc pleurodesis before treatment due to the absence of pleural effusion. Their mPFS and mOS were 6.7 (IQR: 4.4; 9.2) and 13.8 months (IQR: 7.4; 27.1), respectively. In this group, 27 patients achieved PR (41.5%), 29 SD (44.6%), and 9 PD (13.9%).

**Figure 1:**
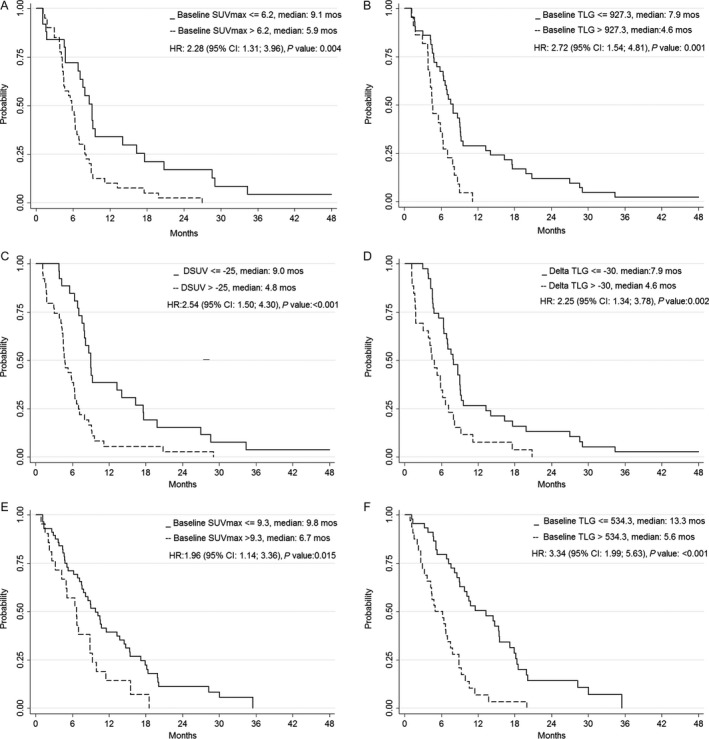
Progression‐free survival stratified for (A) baseline SUV_max_ value in patients without talc pleurodesis (B) baseline TLG value in patients without talc pleurodesis; (C) ∆SUV in patients without talc pleurodesis; (D) ∆TLG in patients without talc pleurodesis; (E) baseline SUV_max_ value in patients with talc pleurodesis; (F) baseline TLG value in patients with talc pleurodesis.

**Figure 2:**
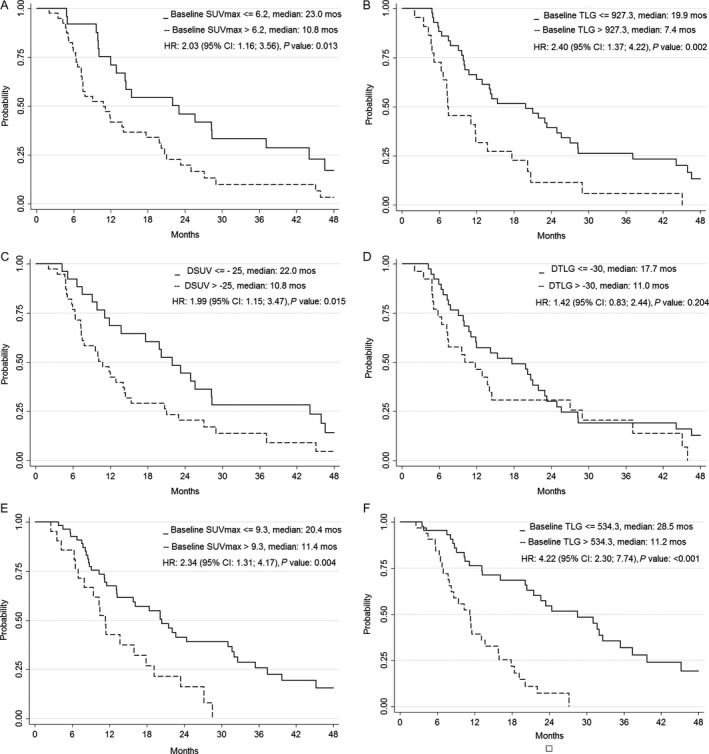
Overall survival stratified for (A) baseline SUVmax value in patients without talc pleurodesis (B) baseline TLG value in patients without talc pleurodesis; (C) ∆SUV in patients without talc pleurodesis; (D) ∆TLG in patients without talc pleurodesis; (E) baseline SUVmax value in patients with talc pleurodesis; (F) baseline TLG value in patients with talc pleurodesis.

On univariate analysis, tumor histology, SUV_max_ at baseline, TLG at baseline and ∆SUV showed a statistically significant association with both PFS and OS, while ∆TLG showed a statistically significant association with PFS (Table 3). EORTC score was a prognostic factor for OS only. The recursive analysis identified indicative cut‐offs of 6.2 for SUV_max_ and 927.3 for TLG, while corresponding cut‐offs for ∆SUV and ∆TLG were −27.8% and −34.97%, respectively. These last two values were similar to previously published data; therefore, we applied a SUV reduction of ≥25% (∆SUV ≥ 25%) and a TLG reduction of ≥30% (∆TLG ≥ 30%) as reference cut‐off values 22, 23. PET parameters categorized according to cut‐off values were significantly associated with outcome, except ∆TLG that showed a statistically significant association with PFS only (Figs. 1 and 2).

**Table 3:** Univariate survival analysis in patients without talc pleurodesis and in patients treated with talc pleurodesis

Characteristics	NO TALC PLEURODESIS						TALC PLEURODESIS					
	PFS			OS			PFS			OS		
	HR	95% CI	*P* value	HR	95% CI	*P* value	HR	95% CI	*P* value	HR	95% CI	*P* value
Sex (M vs. F)	1.39	0.83; 2.32	0.206	1.81	1.05; 3.12	0.033	0.58	0.33; 1.02	0.057	0.82	0.46; 1.46	0.499
ECOG PS (1 and 2 vs. 0)	1.60	0.97; 2.66	0.068	1.65	0.96; 2.84	0.070	2.11	1.27; 3.49	0.004	2.62	1.51; 4.56	<0.001
Histology (NE vs. E)	2.73	1.42, 5.27	0.003	2.25	1.14; 4.43	0.019	1.55	0.78; 3.05	0.209	2.62	1.28; 5.34	0.008
EORTC score (poor vs. good)	1.61	0.97; 2.67	0.066	1.74	1.02; 2.96	0.043	1.30	0.79; 2.13	0.309	1.53	0.90; 2.59	0.115
SUVmax baseline (for every 1 unit)	1.10	1.04; 1.16	<0.001	1.08	1.03; 1.14	0.004	1.10	1.03; 1.17	0.003	1.12	1.05; 1.19	<0.001
TLG baseline (for every 10 units)	1.00	1.00; 1.00	0.003	1.00	1.00; 1.00	0.020	1.00	1.00; 1.00	<0.001	1.01	1.00; 1.01	<0.001
ΔSUV (for every 10 units)	1.09	1.04; 1.15	<0.001	1.05	1.01; 1.11	0.031	1.01	0.95; 1.07	0.757	0.96	0.90; 1.03	0.247
ΔTLG (for every 10 units)	1.03	1.02; 1.05	<0.001	1.00	1.00; 1.02	0.671	1.01	0.99; 1.02	0.627	1.00	0.99; 1.02	0.721

ECOG, Eastern Cooperative Oncology Group; PS, performance status; NE, not epithelioid; E, epithelioid; EORTC = European Organization for Research and Treatment of Cancer; SUV_max_, maximum standardized uptake value; TLG, total lesion glycolysis; ∆TLG, percentage change in TLG between baseline PET and interim PET after two cycles of therapy; ∆SUV, percentage change in SUV_max_ between baseline PET and interim PET after two cycles of therapy.

On multivariate analysis, all PET parameters considered at baseline, that is, SUV_max_ (*P* = 0.030) and TLG (*P* = 0.047), and after two cycles of chemotherapy, that is, ∆SUV (*P* = 0.028) and ∆TLG (*P* = 0.049), were significantly associated with PFS. On the other hand, only SUV_max_ (*P* = 0.005) was significantly associated with OS. Upon combining the two PET parameters as variation after two cycles of chemotherapy, patients showing both ∆SUV (∆SUV ≥ 25%) and ∆TLG (∆TLG ≥ 30%) responses had a significant reduction in the risk of disease progression (HR: 0.31, 95% CI: 0.17; 0.57, *P* < 0.001) and death (HR: 0.52, 95% CI: 0.28; 0.98, *P* = 0.044).

### Patients treated with talc pleurodesis

Seventy‐seven patients received talc pleurodesis before chemotherapy due to the presence of pleural effusion. Their mPFS and mOS were 8·9 (IQR: 4.7; 15.4) and 17.9 (IQR: 8.8; 31.7) months, respectively. Overall, 21 patients achieved PR (27.3%), 44 SD (57.1%), and 12 PD (15.6%).

Baseline values of PET parameters in patients treated with pleurodesis were not significantly different from those in patients not treated with pleurodesis (*P* = 0.863 and *P* = 0.389 for SUV_max_ and TLG, respectively). Moreover, baseline PET parameters did not differ significantly from those evaluated after two cycles of chemotherapy (*P* = 0.805 and *P* = 0.343 for SUV_max_ and TLG, respectively).

On univariate analysis, ECOG PS, SUV_max_ at baseline and TLG at baseline showed a statistically significant association with both PFS and OS, whereas histology was associated with OS only (Table 3). The recursive analysis identified indicative cut‐offs for SUV_max_ (9.25) and TLG (534.3) at baseline that distinguished patients with a different outcome (Figs. 1 and 2). On multivariate analysis, baseline TLG (*P* < 0.001) had a significant association with both PFS and OS, whereas ECOG PS and tumor histology were associated only with PFS and OS, respectively.

None of the variations after two cycles of chemotherapy (i.e., ∆SUV and ∆TLG) considered in continuous form or using the cut‐off percentages cited above showed a significant association with PFS or OS.

## Discussion

FDG‐PET has been increasingly used in MPM for staging and monitoring tumor response to chemotherapy. In fact, preliminary observations suggested that MPM avidity for FDG might be regarded as a surrogate marker of tumor biology with a prognostic significance, while therapy‐induced changes in FDG uptake might predict response and patient outcome early in the course of therapy 25.

Flores et al. incorporated SUV_max_ into a prognostic model with stage and histology, observing that a SUV_max_ value >10 was associated with poor prognosis 26. Similarly, SUV_max_ was an independent predictor of survival in two other patient series, with cut‐off values of 10.7 and 5, respectively 10, 27. In contrast, Nowak et al. reported that FDG‐PET volumetric parameters significantly predicted survival, whereas SUV_max_ did not 9. In particular, baseline TGV was included in a nomogram of pretreatment prognostic factors for MPM. Recently, Klablasta et al. confirmed TLG and histology as independent prognostic factors, whereas Hooper et al. observed baseline TGV as an independent predictor of worse OS in this disease 11, 12. Moreover, Kodota et al. 28 observed that the baseline level of SUV_max_ could identify also the subgroup having the worse prognosis among patients with epithelial histology.

In our cohort of patients not receiving pleurodesis, a SUVmax ≥ 6.2 at baseline was significantly associated with a poor prognosis, in agreement with literature data 10, 26, 27. Although we applied the same quantification method as used by Nowak et al., at multivariate analysis, only baseline SUVmax showed a statistically significant correlation with OS, whereas TLG did not 9.

We hypothesize that SUVmax could identify the most aggressive tumor clones that drive the prognosis of the disease. Probably, this sign of malignity is underrated in TLG analysis due to the algorithm that calculates this value 29. This sort of calculation could therefore obscure the significance of focal uptake identified with SUVmax. Conversely, because TLG constitutes an overall estimate of tumor (metabolic) burden, it might be more suitable for response assessment rather than survival prognostication. In clinical practice, these data suggest that SUVmax could be sufficient to determine the prognosis of patients not submitted to pleurodesis.

On the other hand, in our cohort of patients treated with pleurodesis, baseline TLG was a strong independent prognostic factor for PFS and OS, regardless of the inflammatory effects induced by pleurodesis itself. In particular, patients receiving pleurodesis and having a baseline TLG ≤ 534.3 showed a mOS significantly longer than patients with a TLG > 534.3. These results are in agreement with the data of Hooper et al., who reported that baseline TGF predicted the prognosis independently of talc pleurodesis, and with the data of Nowak et al., who observed that baseline TGV remained predictive of survival in patients with previous pleurodesis, independently of histology 9, 12. Taken together, these data support the prognostic role of quantitative PET parameters even in patients treated with pleurodesis, at least at baseline.

Several preliminary studies have explored the role of metabolic response evaluated by FDG‐PET in MPM patients treated with pemetrexed‐based chemotherapy who have not received talc pleurodesis. In these studies, semiquantitative (SUV_max_) and quantitative analyses (MTV, TGV or TLG) were applied by computing variations in areas of FDG accumulation at different time points during treatment 11, 12, 13, 14, 15, 16, 17, 18. In a previous study by our group, a 25% decrease in SUV_max_ correlated with improved time to progression (14 months vs. 7 months in nonresponders) 13. However, considering that MPM is often diffuse and heterogeneous, several authors have postulated that SUV_max_, as a single‐pixel parameter, may not be representative of changes within the entire tumor following chemotherapy 14, 30. Veit‐Haibach et al. reported that a TGV reduction obtained after three cycles of chemotherapy was predictive of response as determined by RECIST criteria 15. Both TGV reduction and CT scan response were associated with improved survival, whereas SUV_max_ and SUV_mean_ were not, suggesting that volumetric PET measurements of tumor uptake may be more accurate than SUV_max_. Evidence of response was reported by Francis et al. as early as after one cycle of chemotherapy using a quantitative semiautomated volume‐based FDG‐PET analysis able to obtain the TGV 14. All the reported data, although obtained in small cohorts, suggest that in MPM patients treated with chemotherapy, an early reduction in FDG uptake can be correlated with patient outcome, in particular when talc pleurodesis is not performed. By contrast, Hooper et al. observed that change in interval TGV (baseline/after two cycles of chemotherapy) did not predict OS or chemotherapy response on CT scan 12. In particular, analyzing 33 out of 41 (80%) MPM patients classified as metabolic responders on interval PET‐CT (30% or greater fall in TGV), they did not observe a significant difference between the metabolic responders and nonmetabolic responders group in terms of time to progression on interval CT scan at 2 months (after three cycles of chemotherapy).

In our cohort of patients not treated with talc pleurodesis, ∆SUV and ∆TLG after two cycles of chemotherapy were significantly correlated with PFS, suggesting their predictive role in response assessment. Recursive analysis on our cohort of patients identified −27.8% and −34.97% as the cut‐off percentages of metabolic response in terms of reduction in SUV and TLG, respectively. From these data, in agreement with previously published data for other tumors, we postulate that reductions of ≥25% in SUV and ≥30% in TLG (i.e., ∆SUV ≥ 25% and ∆TLG ≥ 30%) might have a role in defining metabolic response 23, 24. The added value of the assessment of metabolic response on PET, as previously reported by our group, could reside in its ability to predict outcome in MPM patients who show SD on CT scan 13, 19. When ∆SUV and ∆TLG were combined, the correlation with PFS improved, suggesting that while ∆SUV alone could be sufficient in clinical practice, the use of both parameters could be more appropriate in clinical trials, when the aim is to test a new treatment.

In patients treated with talc pleurodesis, neither ∆SUV nor ∆TLG showed a significant correlation with PFS or OS, suggesting that FDG signal in these patients is not reliable in the presence of an important inflammatory process. Potentially, the FDG uptake due to inflammation could mask the tumor uptake, particularly in the presence of tumors with low baseline FDG‐avidity. In fact, regardless of talc pleurodesis, either ∆SUV or ∆TLG evaluations remain challenging in patients with low SUVmax at baseline. New radiopharmaceuticals under investigation may overcome the limitations demonstrated by FDG in this setting 31.

In conclusion, this trial confirms the prognostic role of baseline FDG‐PET in a large series of MPM patients treated with first‐line pemetrexed‐based chemotherapy. Moreover, the use of a SUV_max_ reduction ≥25% and a TLG reduction ≥30% as cut‐off values for the definition of metabolic response after two cycles of chemotherapy, confirms the role of FDG‐PET in predicting disease outcome and treatment response in patients not submitted to talc pleurodesis.

## Conflict of Interest

All the authors indicate no financial or other interest that is relevant to the subject matter under consideration in this article.

## References

[1] Robinson, B. M. 2012 Malignant pleural mesothelioma: an epidemiological perspective. Ann. Cardiothorac Surg. 1:491–496. 23977542 10.3978/j.issn.2225-319X.2012.11.04 PMC3741803

[2] Byrne, M. J. , and A. K. Nowak . 2004 Modified RECIST criteria for assessment of response in malignant pleural mesothelioma. Ann. Oncol. 15:257–260. 14760119 10.1093/annonc/mdh059

[3] Ceresoli, G. L. , A. Chiti , P. A. Zucali , et al. 2007 Assessment of tumor response in malignant pleural mesothelioma. Cancer Treat. Rev. 33:533–541. 17764849 10.1016/j.ctrv.2007.07.012

[4] Armato, III S. G. , J. L. Ogarek , A. Starkey , et al. 2006 Variability in mesothelioma tumor response classification. AJR Am. J. Roentgenol. 186:1000–1006. 16554570 10.2214/AJR.05.0076

[5] Oxnard, G. R. , S. G. Armato III , and H. L. Kindler . 2006 Modeling of mesothelioma growth demonstrates weakness of current response criteria. Lung Cancer 52:141–148. 16530882 10.1016/j.lungcan.2005.12.013

[6] Larson, S. M. , Y. Erdi , T. Akhurst , et al. 1999 Tumor treatment response based on visual and quantitative changes in global tumor glycolysis using PET‐FDG imaging. The visual response score and the change in total lesion glycolysis. Clin. Positron Imaging 2:159–171. 14516540 10.1016/s1095-0397(99)00016-3

[7] Herndon, J. E. , M. R. Green , A. P. Chahinian , et al. 1998 Factors predictive of survival among 337 patients with mesothelioma treated between 1984 and 1994 by the Cancer and Leukemia Group B. Chest 113:723–731. 9515850 10.1378/chest.113.3.723

[8] Curran, D. , T. Sahmoud , P. Therasse , et al. 1998 Prognostic factors in patients with pleural mesothelioma: the European Organization for Research and Treatment of Cancer experience. J. Clin. Oncol. 16:145–152. 9440736 10.1200/JCO.1998.16.1.145

[9] Nowak, A. K. , R. J. Francis , M. J. Phillips , et al. 2010 A noverl prognostic model for malignant mesothelioma incorporating quantitative FDG‐pet imaging and clinical parameters. Clin. Cancer Res. 16:2409–2417. 20371686 10.1158/1078-0432.CCR-09-2313

[10] Abakay, A. , H. Komek , O. Abakay , et al. 2013 Relationship between 18 FDG PET‐CT findings and survival of 177 patients with malignant pleural mesothelioma. Eur. Rev. Med. Pharmacol. Sci. 17:1233–1241. 23690193

[11] Klabatsa, A. , S. Chicklore , S. Barrington , et al. 2014 The association of 18F‐FDG PET/CT parameters with survival in malignant pleural mesothelioma. Eur. J. Nucl. Med. Mol. Imaging 41:276–282. 24057459 10.1007/s00259-013-2561-1

[12] Hooper, C. E. , I. D. Lyburn , J. Searle , et al. 2015 The south west area Mesothelioma and Pemetrexed trial: a multicentre prospective observational study evaluating novel markers of chemotherapy response and prognostication. Br. J. Cancer 112:1175–1182. 25756396 10.1038/bjc.2015.62 PMC4385956

[13] Ceresoli, G. L. , A. Chiti , P. A. Zucali , et al. 2006 Early evaluation in malignant pleural mesothelioma by positron emission tomography with [18F] fluorodeoxyglucose. J. Clin. Oncol. 24:4587–4593. 17008700 10.1200/JCO.2006.06.8999

[14] Francis, R. J. , M. J. Byrne , A. A. van der Schaaf , et al. 2007 Early prediction of response to chemotherapy and survival in malignant pleural mesothelioma using a novel semiautomated 3‐dimensional volume‐based analysis of serial 18F‐FDG PET scans. J. Nucl. Med. 48:1449–1458. 17704250 10.2967/jnumed.107.042333

[15] Veit‐Haibach, P. , N. G. Schaefer , H. C. Steinert , et al. 2010 Combined FDG‐PET/CT in response evaluation of malignant pleural mesothelioma. Lung Cancer 67:311–317. 19482372 10.1016/j.lungcan.2009.04.015

[16] Lee, H. Y. , S. H. Hyun , K. S. Lee , et al. 2010 Volume‐based parameter of 18F‐FDG PET/CT in malignant pleural mesothelioma: prediction of therapeutic response and prognostic implications. Ann. Surg. Oncol. 17:2787–2794. 20461469 10.1245/s10434-010-1107-z

[17] Genestreti, G. , A. Moretti , S. Piciucchi , et al. 2012 FDG PET/CT response evaluation in malignant pleural mesothelioma patients treated with talc pleurodesis and chemotherapy. J. Cancer 3:241–245. 22670158 10.7150/jca.2586 PMC3366479

[18] Schaefer, N. G. , P. Veit‐Haibach , J. D. Soyka , et al. 2012 Continued pemetrexed and platin‐based chemotherapy in patients with malignant pleural mesothelioma (MPM): value of 18F‐FDGPET/CT. Eur. J. Radiol. 81:e19–e25. 21129871 10.1016/j.ejrad.2010.11.006

[19] Lopci, E. , P. A. Zucali , G. L. Ceresoli , et al. 2015 Quantitative analyses at baseline and interim PET evaluation for response assessment and outcome definition in patients with malignant pleural mesothelioma. Eur. J. Nucl. Med. Mol. Imaging 42:667–675. 25403555 10.1007/s00259-014-2960-y

[20] Boellaard, R. , R. Delgado‐Bolton , W. J. Oyen , et al. 2015 FDG PET/CT: EANM procedure guidelines for tumour imaging: version 2.0. Eur. J. Nucl. Med. Mol. Imaging 42:328–354. 25452219 10.1007/s00259-014-2961-x PMC4315529

[21] Boucek, J. , R. J. Francis , and A. J. Green . 2005 Automated approach to identification and quantitation of tumor volumes in chemotherapy monitoring using FDG PET. J. Nucl. Med. 46(suppl):464P(abstr).15750160

[22] Lopci, E. , P. Zucali , L. Giordano , et al. 2014 Validation of liver‐based quantitative analysis on PET for response assessment in patients with malignant pleural mesothelioma. J. Nucl. Med. 55(suppl 1):458(abstr).

[23] Young, H. , R. Baum , U. Cremerius , et al. 1999 Measurement of clinical and subclinical tumor response using [18F]‐fluorodeoxyglucose and positron emission tomography: review and 1999 EORTC recommendations. Eur. J. Cancer 35:1773–1782. 10673991 10.1016/s0959-8049(99)00229-4

[24] Wahl, R. L. , H. Jacene , Y. Kasamon , and M. A. Lodge . 2009 From RECIST to PERCIST: evolving considerations for PET response criteria in solid tumors. J. Nucl. Med. 50:122S–150S. 19403881 10.2967/jnumed.108.057307 PMC2755245

[25] Strorto, G. , E. Nicolai , and M. Salvatore . 2009 [18F]FDG‐PET/CT for early monitoring of tumor response: when and why. Q. J. Nucl. Med. Mol. Imaging 53:167–180. 19293765

[26] Flores, R. M. , T. Akhurst , M. Gonen , et al. 2006 Positron emission tomography predicts survival in malignant pleural mesothelioma. J. Thorac. Cardiovasc. Surg. 132:763–768. 17000285 10.1016/j.jtcvs.2006.03.068

[27] Gerbaudo, V. H. , M. Mamede , B. Trotman‐Dickenson , H. Hatabu , and D. J. Sugarbaker . 2011 FDG PET/CT patterns of treatment failure of malignant pleural mesothelioma: relationship to histologic type, treatment algorithm, and survival. Eur. J. Nucl. Med. Mol. Imag. 38:810–821. 10.1007/s00259-010-1704-x 21210110

[28] Kadota, K. , S. S. Kachala , J. Nitadori , et al. 2012 High SUVmax on FDG‐PET indicates pleomorphic subtype in epithelioid malignant pleural mesothelioma: supportive evidence to reclassify pleomorphic as non‐epithelioid histology. J. Thorac. Oncol. 7:1192–1197. 22617244 10.1097/JTO.0b013e3182519d96 PMC3691682

[29] Fathinul, F. , A. J. Nordin , and W. F. Lau . 2013 18[F]FDG‐PET/CT is a useful molecular marker in evaluating tumour aggressiveness: a revised understanding of an in‐vivo FDG‐PET imaging that alludes the alteration of cancer biology. Cell Biochem. Biophys. 66:37–43. 22790883 10.1007/s12013-012-9395-5

[30] Boucek, J. A. , R. J. Francis , C. G. Jones , et al. 2008 Assessment of tumor response with 18F‐fluorodeoxyglucose positron emission tomography using three‐dimensional measures compared to SUVmax – a phantom study. Phys. Med. Biol. 53:4213–4230. 18653927 10.1088/0031-9155/53/16/001

[31] Ceresoli, G. L. , A. Chiti , and A. Santoro . 2007 11C‐labeled methionine and evaluation of malignant pleural mesothelioma. N. Engl. J. Med. 357:1982–1984. 17989397 10.1056/NEJMc071823

